# A genetic researcher’s devil’s dilemma: Warn relatives about their genetic risk or respect confidentiality agreements with research participants?

**DOI:** 10.1186/s12910-021-00721-4

**Published:** 2021-11-23

**Authors:** Lieke M. van den Heuvel, Els L. M. Maeckelberghe, M. Corrette Ploem, Imke Christiaans

**Affiliations:** 1 Department of Clinical Genetics, Amsterdam UMC (Location AMC)/University of Amsterdam, Meibergdreef 9, 1105 AZ Amsterdam, the Netherlands; 2 Netherlands Heart Institute, Utrecht, the Netherlands; 3 Department of Genetics, University Medical Center Utrecht/University Utrecht, Utrecht, the Netherlands; 4 Institute for Medical Education, University Medical Center Groningen/Groningen University, Groningen, the Netherlands; 5 Department of Public Health, Amsterdam UMC (Location AMC)/University of Amsterdam, Amsterdam, the Netherlands; 6 Department of Genetics, University Medical Center Groningen/Groningen University, Groningen, the Netherlands

**Keywords:** Family communication research, Genetic risk, Duty to warn, Informing at-risk relatives

## Abstract

**Background:**

With advances in sequencing technologies, increasing numbers of people are being informed about a genetic disease identified in their family. In current practice, probands (the first person in a family in whom a genetic predisposition is identified) are asked to inform at-risk relatives about the diagnosis. However, previous research has shown that relatives are sometimes not informed due to barriers such as family conflicts. Research on family communication in genetic diseases aims to explore the difficulties encountered in informing relatives and to identify ways to support probands in this.

**Main body:**

Research on family communication may also reveal that participants did not inform their relatives about the risk of a serious genetic condition, even when preventive and treatment options are available. Researchers may then face a dilemma: Do they need to warn at-risk relatives about the finding? Or do they keep silent due to prior confidentiality agreements with study participants?

**Conclusions:**

We believe that the absolute confidence promised to research participants outweighs the interests of their relatives, even though it can be claimed that relatives at risk of a genetic disease do, in principle, have a right to know information collected about their health. Not respecting confidentiality agreements could cause distrust between researchers and research participants and possibly harm the relationship between probands and relatives. Relatives' health interests can still be taken into account without jeopardizing participant trust, by considering alternative scenarios, including sharing general study findings on the barriers participants experience with their healthcare professionals and by offering participants psychosocial support for family communication.

## Background

Over the past few decades, new technologies in the field of genetics and genomics have led to an increasing number of individuals being identified as at risk for a hereditary disease. Currently, the proband (the first person in a family in whom the genetic predisposition is identified) is asked to inform at-risk relatives about the hereditary disease and the possibility of predictive DNA testing. However, as the result of several barriers, relatives are sometimes not informed. In this article, we focus on the issue of what to do when a researcher discovers information that could potentially ‘rescue’ a research participant’s relatives (Table 1).

**Table 1:** (Fictional) case description

“A disease-causing genetic variant in the BRCA1 gene is identified in a 45-year-old female diagnosed with breast cancer. She is asked to participate in a study on the approach to informing at-risk relatives in hereditary breast and ovarian cancer, which generally follow an autosomal dominant inheritance pattern. During this study, the participant is asked whether she has informed her relatives. When she received the DNA test result, she had expressed the intention to inform her daughters. During the study, however, the participant informs the researcher that she felt unable to inform her two daughters (25 and 27 years old). She tried to tell them multiple times but felt that she could not find the right moment. The participant explains that she is afraid of upsetting herself and of burdening her daughters with such sensitive information and she thinks she should wait until her daughters are in their thirties and have their lives sorted out a bit more. In this particular setting, the researcher faces an ethical dilemma: the participant’s daughters are at an age when regular screening for breast cancer is advised when there is an increased risk and leaving them uninformed could potentially harm them. However, warning them would significantly violate the confidentiality agreement with the research participant.”	

We start by briefly describing the clinical context of family communication in genetics and the circumstances under which such dilemmas for researchers investigating this subject could arise. We then discuss current legal and ethical principles that can be used in addressing the responsibilities of researchers towards their research participants and, more specifically, towards participant’s relatives, as well as discussing factors to consider in disclosure of hereditary risk information to relatives. We conclude that confidentiality agreements with the research participant should be respected as much as possible and ultimately outweigh the potential harm that could be prevented by directly informing at-risk relatives.

### Researchers facing a dilemma

#### Family communication in clinical genetics

In autosomal dominant disease, all first-degree relatives have a 50% chance of inheriting the genetic predisposition to the disease. Predictive DNA testing can determine if relatives have inherited the predisposition, and non-carriers can then be reassured that they do not carry the predisposition, while carriers can be assisted in making informed health decisions. In certain diseases such as inherited cardiac diseases and hereditary types of cancer, prevention and treatment options are available that may decrease morbidity and mortality [1, 2]. Furthermore, a DNA test result may be informative for life and reproductive choices [2]. However, knowledge about the increased risk of developing a hereditary disease may also have profound psychological impact, including anxiety, uncertainty and depressive symptoms, and potentially negative consequences for insurance coverage [3, 4].

In current clinical practice, the genetic healthcare professional asks the proband to inform at-risk relatives about the genetic disease and the possibility of predictive DNA testing and/or advice over clinical screening, and this approach is often supported by a family letter provided by the genetic healthcare professional [5]. This current procedure, however, is not as effective as genetic healthcare professionals had hoped, as the uptake of genetic counselling is not considered optima with only half of at-risk relatives attending genetic counselling [6, 7]. Moreover, many barriers to informing at-risk relatives have been described, such as family conflicts, the proband not wanting to burden at-risk relatives and the complexity of genetic information [5, 8–10]. These barriers may result in certain relatives not being informed, or being incorrectly informed, which hampers informed decision-making about predictive DNA testing [9].

To enable relatives in making a well-informed decision about predictive DNA testing, healthcare professionals aim to improve communication between probands and relatives. Healthcare professionals and researchers in the United Kingdom and the Netherlands have suggested that a (slightly) more active approach to informing at-risk relatives might be effective in ensuring that at-risk relatives are informed [1, 7, 11]. Genetic healthcare professionals in the Netherlands also seem to adopt a more active role in informing at-risk relatives by having a standard follow-up contact with the proband several months after test result disclosure and by suggesting a direct contact approach for relatives in case the proband is unable or unwilling to inform [12].

#### Research on family communication in clinical genetics

Research on family communication in the field of clinical genetics aims to explore the difficulties encountered in informing at-risk relatives, to identify factors that may influence this family communication and to find ways to support probands in this process. These studies can involve collection of data about which relatives are being informed, but even more importantly, whether certain relatives are not being informed. However, data collection in these studies may result in researchers becoming aware of familial information that could be of great importance to relatives’ health, especially for diseases for which preventive or treatment options are available.

#### The dilemma

Researchers may then experience a dilemma. In Table 1, we describe a case in which such a dilemma occurs. A female research participant reveals to the researcher that she did not inform her at-risk daughters about the hereditary breast-ovarian cancer diagnosed in their family. The researcher thus faces a dilemma: Respect the participant’s wishes and her decision to not yet inform her daughters? Or ensure that the daughters are informed about their risk of developing breast cancer at an early age? Similar dilemmas that involve an ethical conflict between breaking confidentiality agreements with a study participant and trying to prevent harm to at-risk relatives or to other people have also been described in research on the prevention of child abuse, child safety and HIV [13–15].

## Main body

### Conflicting duties

#### Context of clinical care

To set the stage, we need to discuss the responsibility of healthcare professionals regarding a proband’s at-risk relatives, as it could be informative for a similar responsibility of researchers regarding the relatives of study participants. The principal duty of healthcare professionals is to provide appropriate care to the person who sought their help and do all they can to benefit the patient (in each situation), to promote their patient’s autonomy and to do no harm. Moreover, healthcare professionals have a confidential (physician–patient) relationship with probands in which all information shared or discussed is protected by the duty of medical confidentiality [16]. In most countries, healthcare professionals do not owe substantial duties to individuals who did not seek their help, and it is therefore not their primary duty to (also) serve the health interests of their patient’s relatives [17].

However, considering the familial nature of genetic information, it is of significance to the proband’s relatives, and healthcare professionals could thus also be thought to have some responsibility towards at-risk relatives [18]. After all, non-disclosure by a proband could, in certain cases, lead to serious harm for at-risk relatives. Depending on the situation, healthcare professionals may therefore have a (moral) duty to prevent harm to the health of the proband’s relatives [11, 19–21].

In clinical genetic practice, this duty ‘to prevent harm’ can be either operationalized by informing probands about their relatives’ genetic risks and the possibility of predictive DNA testing or by directly disclosing this information to the relatives (in principle with the patient’s permission). The first (weaker) form of communication, which is current clinical practice, protects the patient’s autonomy and confidentiality but leans on the patient’s willingness and capacity to inform at-risk relatives [11, 19, 21]. When, in exceptional cases, the proband is unable or unwilling to inform at-risk relatives, and refuses to consent to the healthcare professional informing them directly, the genetic information will not be disclosed to relatives unless their interests clearly outweigh the proband’s interest in keeping their genetic data confidential [11, 19–21]. In the latter situation, the healthcare professional finds themself in a ‘conflict of duties’ between the duty to protect patient confidentiality and the duty to prevent serious harm in relatives [11, 20, 21].

#### Context of research

Whereas providing care for their patients is the first responsibility of genetic healthcare professionals, and this responsibility may extend to the proband’s relatives when the disease is familial, the situation is clearly different for researchers. In contrast to genetic healthcare professionals, who have a clear duty of care towards their patients, the primary duty of observational researchers is to generate new knowledge by conducting research that respects research participant’s rights [22]. Furthermore, the researcher-participant relationship is in general a short-term relationship, which is – in contrast to the relationship between a healthcare professional and a patient – non-therapeutic. Equally, research participants will primarily expect to contribute to the general interest and not to obtain direct benefits for themselves (unless involved in therapeutic studies). In this specific context, the confidentiality agreements between researchers and their participants play an essential role. For participants, confidentiality will undoubtedly form a crucial condition to entrust their most sensitive information to the researcher. In principle, these agreements leave no room for sharing information collected during research with people outside the research team (‘third parties’), e.g. healthcare professionals or relatives of the study participant [17].

However, just as for healthcare professionals, it is understandable that researchers feel a ‘conflict of duties’ when they encounter information such as undisclosed genetic risk that would be highly significant for relatives. If this information is disclosed in time, harm to these at-risk relatives could be prevented. Nonetheless, informing a participant’s relative about genetic risk conflicts with the participant’s autonomous decision not to inform their relatives.

At the same time, also in the context of research, there can be circumstances in which it may even be expected that researchers share important health information uncovered during the study [22]. This involves situations in which the participants are exposed to an immediate and severe health problem that can be remedied. According to English law, the researchers’ duty ‘to warn’, if any, needs to be ‘fair, just and reasonable’ [17]. We can also refer to international treaty law here, for example the Additional Protocol to the Biomedicine Convention on Biomedical research [23]. According to Article 27 of this Protocol, research findings that are relevant to research participant’s current or future health should be disclosed to them [23].

Moreover, in the context of genetic research, there is an increased probability that a researcher discovers an individual finding that impacts not only the participant, but also their relatives. In that perspective, one could argue that such a duty of care may also apply to proband’s relatives, as also suggested by Ulrich [24]. He argues that, in cases where the research findings involve direct consequences for the research participant’s relatives (as is the case in genomics research), the responsibility to inform also relates to them [24].

### Handling the dilemma: four different scenarios

To find a way to responsibly handle the dilemma discussed in this paper, we examine the pros and cons of four possible scenarios: (1) Scenario 1: The researcher directly warns at-risk relatives; (2) Scenario 2: The researcher informs the research participant’s healthcare professional about uninformed relatives; (3) Scenario 3: The researcher explores potential barriers hampering family communication and provides support, or; (4) Scenario 4: The researcher informs the healthcare professional involved of general study findings, including the presence of uninformed relatives.

#### Scenario 1: Researcher directly warns at-risk relatives

A scenario for this dilemma could be that the researcher decides to directly warn the relatives who are unaware of the genetic risks discovered during the study. This could be considered justifiable from the perspective that researchers have, as concluded in “Context of research” section, some responsibility towards their research participant and possibly, in case of genetic disease, also towards at-risk relatives. By warning the latter group, researchers may promote their health and well-being. Particularly for diseases for which preventive or treatment options are available, researchers may consider not informing at-risk relatives as a potentially significant harm. Furthermore, by informing participant’s relatives directly, the researcher can be sure that at-risk relatives are aware of their genetic risk. This also allows researchers to meet any individual’s right to know any information collected about their health (unless they prefer not to know such information), as outlined in article 26 of Additional Protocol to the Biomedicine Convention on Biomedical research [23].

However, it is not just relatives’ right to know information about important health risks that is at stake here. The confidentiality agreements made with research participants and participant autonomy should also be taken into account, and both are protected by the Declaration of Helsinki [25]. In order to enable responsible decision-making about participation, including on the issue of confidentiality and privacy, an informed consent procedure is in place to inform study participants about all the details of the study. Study participation thus holds the expectation that personal information collected during the research will not be shared with others without the participant’s explicit permission [26]. Informing relatives that they are at risk without informing the participant about such a warning could easily undermine research participant’s trust and the safe research environment. This would harm crucial relationships, primarily that between research participant and researcher, but possibly also that between participant and healthcare professional. In the end, it could be science that ‘pays the bill’ for this harm, in that a climate of distrust about how research is performed could lead to reduced participation in research. Furthermore, researchers may not be healthcare professionals themselves and thus not able to communicate medical information. Moreover, warning at-risk relatives interferes with the autonomous decision of probands not to inform their relatives, a decision that they took on the basis of their own specific considerations. It should be noted that both researchers and healthcare professionals are often unaware of family dynamics and potential valid and informed decisions of participants to not inform at-risk relatives. Therefore, they are unable to take these into consideration when warning at-risk relatives [22]. For example, problems in the psychological functioning of individual relatives may be a very valid reason for the proband not to inform them at that moment [22]. As described by Rothstein [27], there is also a so-called duty of loyalty to patients and informed and valid decisions not to disclose should be respected. Finally, apart from ethical and legal objections as previously described, it may not even be possible for researchers to inform certain at-risk relatives, as they may not know their identity or do not have the possibility to collect their contact details.

#### Scenario 2: Researcher informs healthcare professional involved about relatives not informed

In the first scenario, it is researchers who take on the primary responsibility to warn at-risk relatives, largely disregarding the rights and interests of research participants (and their possibly well-considered actions within their family). Considering the duty researchers primarily have, i.e. to generate new knowledge by conducting research in such way that research participant’s rights on confidentiality and privacy are respected, this is unacceptable. One might argue for a less direct approach to responsibility for researchers, i.e. that they inform the treating genetic healthcare professional about the risk for relatives uncovered during the research. The genetic healthcare professional could then decide to fulfil their duty of care towards the relatives, which is somewhat ‘stronger’ than the researcher’s connection and duty (as argued in “Conflicting duties” section). The healthcare professional could do this by first discussing the specific situation with colleagues or, possibly, an ethics committee, and then by planning a follow-up contact and providing support to participants, if needed. Importantly, in case research participants referred to the researcher by the general practitioner or even by self-referral (for example in case of a survey study recruiting participants online), such a scenario would not even be possible.

Nevertheless, when applicable, such an approach would still imply breaking research confidentiality agreements. In this situation, however, research participants should be informed prior to giving informed consent that research findings might be communicated to their healthcare professional when these findings are deemed important (e.g. when certain relatives have not been informed by the participant) [25]. However, this approach could lead to significant bias in the results of research on family communication by deterring potential participants who are not sure they will be able or willing to inform at-risk relatives. Furthermore, if these individuals do participate, the information they share during the study about their experiences and attitudes could potentially be different.

#### Scenario 3: Researcher explores potential barriers hampering family communication and provides support

As a third scenario, researchers may consider not warning relatives but rather exploring exactly what hampers family communication for those research participants who report that they have not informed their relatives, with the aim of offering adequate support. In the case described in Table 1, the research participant may not dare to inform her daughters, or may think, as she reports, that it is not the right time for them to be informed. Her responses may, however, also indicate that she feels the need for support or perhaps even prefers that someone else inform her daughters. The researcher helping to explore the barriers that hamper family communication may potentially help research participants who actually intended to inform at-risk relatives but did not feel able to do so after the result. Furthermore, it would fully respect participant confidentiality and autonomy. However, offering such support in the context of research is not the task of researchers and may confuse participants about the distinction between clinical care and research, which could ultimately result in so-called ‘therapeutic misconception’ [28]. To prevent this, researchers should clearly define and communicate to participants why, and in what context, they are making their suggestions and should limit their help to adequate referral to the participant’s healthcare professional for further support. However, offering support by referring the participant to their healthcare professional during the study may also bias study findings.

#### Scenario 4: Researcher informs healthcare professional about general study findings

In scenario 4, the researchers put the confidentiality of research participants first and respect the proband’s autonomous decision-making. In this case, the researchers share general research findings about uninformed relatives and the problems and barriers that the research participants encountered with the participants’ treating healthcare professional. Healthcare professionals could then consider following-up the patients participating in the study and offering additional assistance to patients who indicate a need for support to overcome the communication problems. Sharing general findings with the healthcare professional involved could be done before publication but after finishing the study (as it is in most research contexts), as this information-sharing process may affect research findings if it occurs while research is still ongoing. Again, in case a research participant was referred to the research project by the general practitioner or self-referred to the researcher, this scenario would not be possible. Of course, in general, informing healthcare professionals about the problems that may arise in family communication and/or the need for follow-up could lead to more relatives being informed about their genetic risk.

## Conclusions

In research investigating the process and outcomes of family communication in the context of hereditary disease, researchers can be confronted with the dilemma of knowing about relatives who have not been informed about a serious health risk. By warning uninformed at-risk relatives, harm could be prevented, especially when prevention and treatment options are available. A researcher may consider warning at-risk relatives directly, or indirectly by informing the treating healthcare professional. However, while the information about genetic risk or about uninformed at-risk relatives would be valuable for, respectively, these relatives and the probands’ healthcare professionals, we feel that the harm caused by breaking confidentiality outweighs the benefits of warning at-risk relatives because it potentially creates distrust between the researcher and study participant and possibly harms the proband and family relationships. In other words, the agreement between researcher and participant to keep all data collected confidential should, in principle, take precedence over the duty to warn relatives. One option to avoid dilemmas like this is to inform participants, prior to their signing the informed consent form, about the possibility that research findings with consequences for their relatives will be directly or indirectly communicated. This, however, is not considered appropriate in the context of research on family communication in hereditary disease as it would create significant bias in the patients who might be inclined to participate as well as in the data collected. This jeaopardizes research integrity, which will be compromised by biased results.

Altogether, to find a way out of our dilemma, we see the most benefit in our fourth scenario: informing the participant’s healthcare professional about the general research results rather than sharing individual findings with specific healthcare professionals. Additionally, researchers could consider referring probands who experience difficulties in informing at-risk relatives to the healthcare professional, as also described in scenario 4. In case a healthcare professional is not involved and thus scenario 4 is not possible, researchers may consider scenario 3 after study completion. This being said, the four scenarios we propose are likely not the only ones. To find the best path forward for all parties, responsible ethical decision making should take place on a case-by-case basis, preferably by discussing a case with colleagues from different multidisciplinary backgrounds (genetics, psychology, ethics, law) or by involving a medical ethics committee.

## Data Availability

Not applicable.
